# Supplementary figures and images for: Abrogation of Stem Loop Binding Protein (Slbp) function leads to a failure of cells to transition from proliferation to differentiation, retinal coloboma and midline axon guidance deficits
Source: PLoS One. 2019 Jan 29;14(1):e0211073. doi: 10.1371/journal.pone.0211073 (PMC6350959; doi:10.1371/journal.pone.0211073)

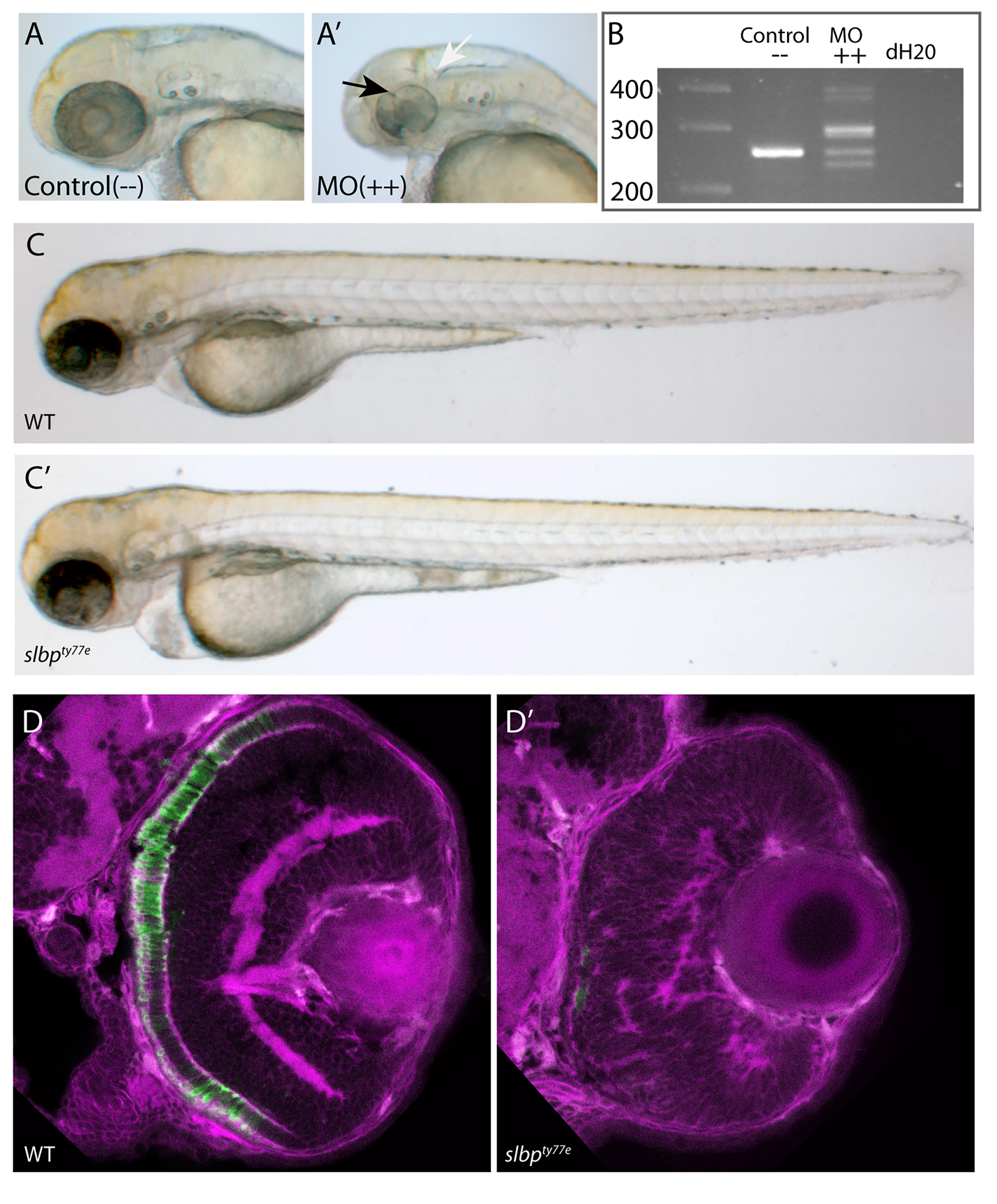

Supplement: S1 Fig — (A-A’) Analysis of live 50hpf Control (A) and Morpholino injected embryos (A’) show that the morphological dent caudal to the MHB (white arrow), indentations in the retina (black arrow) and coloboma are successfully phenocopied. (B) Electrophoresis gel of RT-PCR analysis confirmed that several missplicing events occur as at least five variably sized products were generated (++ lane). (C-C’) Injection of degradation-resistant slbpTT-AA-RFP synthetic RNA into WT(C) and ele(C’)mutants rescues ele phenotype. Note heart oedema still present in some cases (C’). (D-D’) Frontal sections of 3dpf wildtype (D) and slbpty77e (D’) retinas showing anti-γ-tubulin labelled neurites/neuropil (magenta) and Rho4D2-expressing rod photoreceptors (green). (TIF) [file pone.0211073.s001.tif]

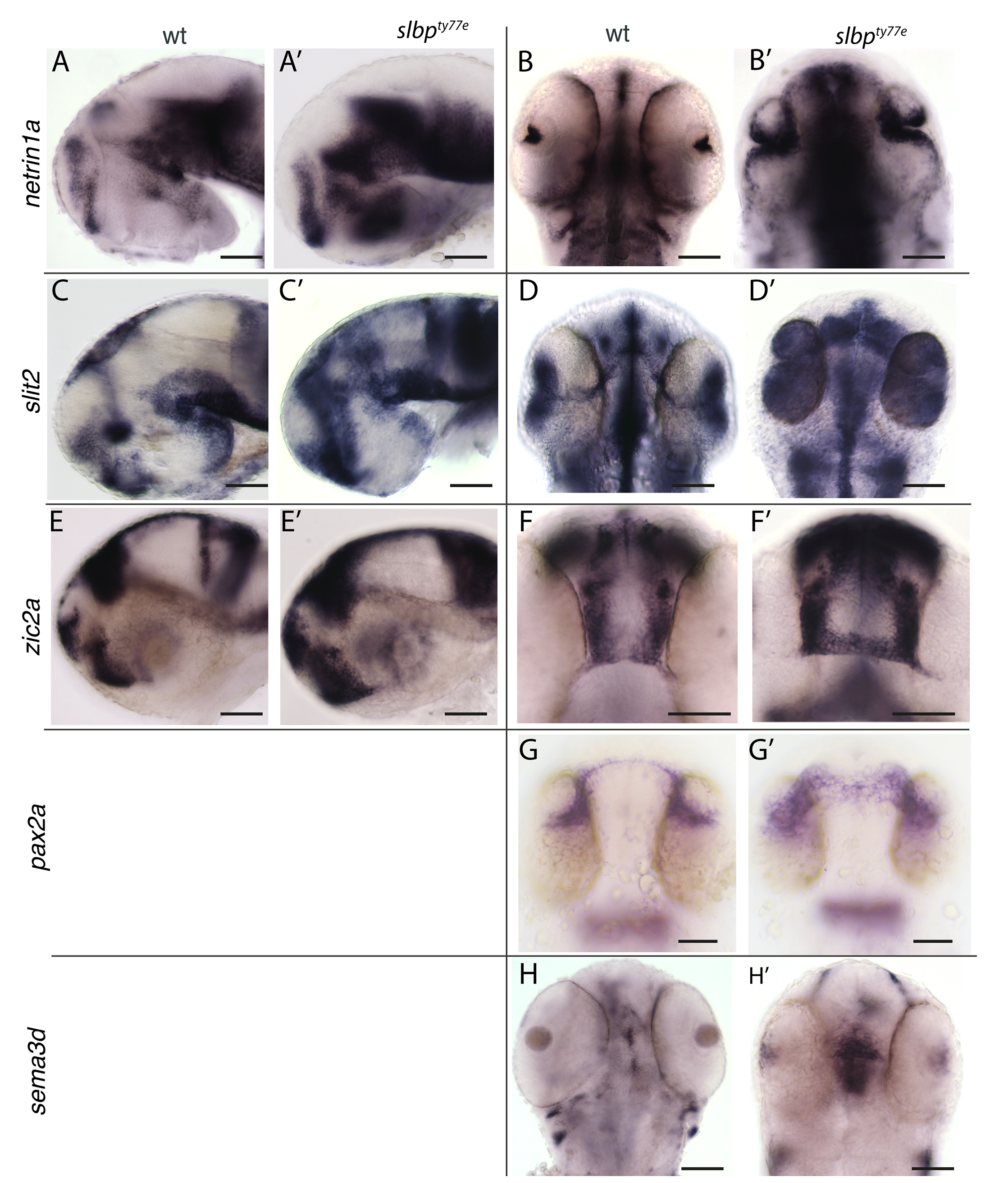

Supplement: S2 Fig — Views of heads/brains of wildtype (A-H) and slbpty77e (A’-H’) embryos showing expression of genes indicated to the left of each row. Genotype is indicated at top of each column. Lateral views (A,A’,C,C’,E,E’); dorsal views (B,B’,D,D’,F,F’,G,G’,H,H’). All embryos are 60hpf apart from G,G’ which are 30hpf. Scale bars: 100μm. (TIF) [file pone.0211073.s002.tif]

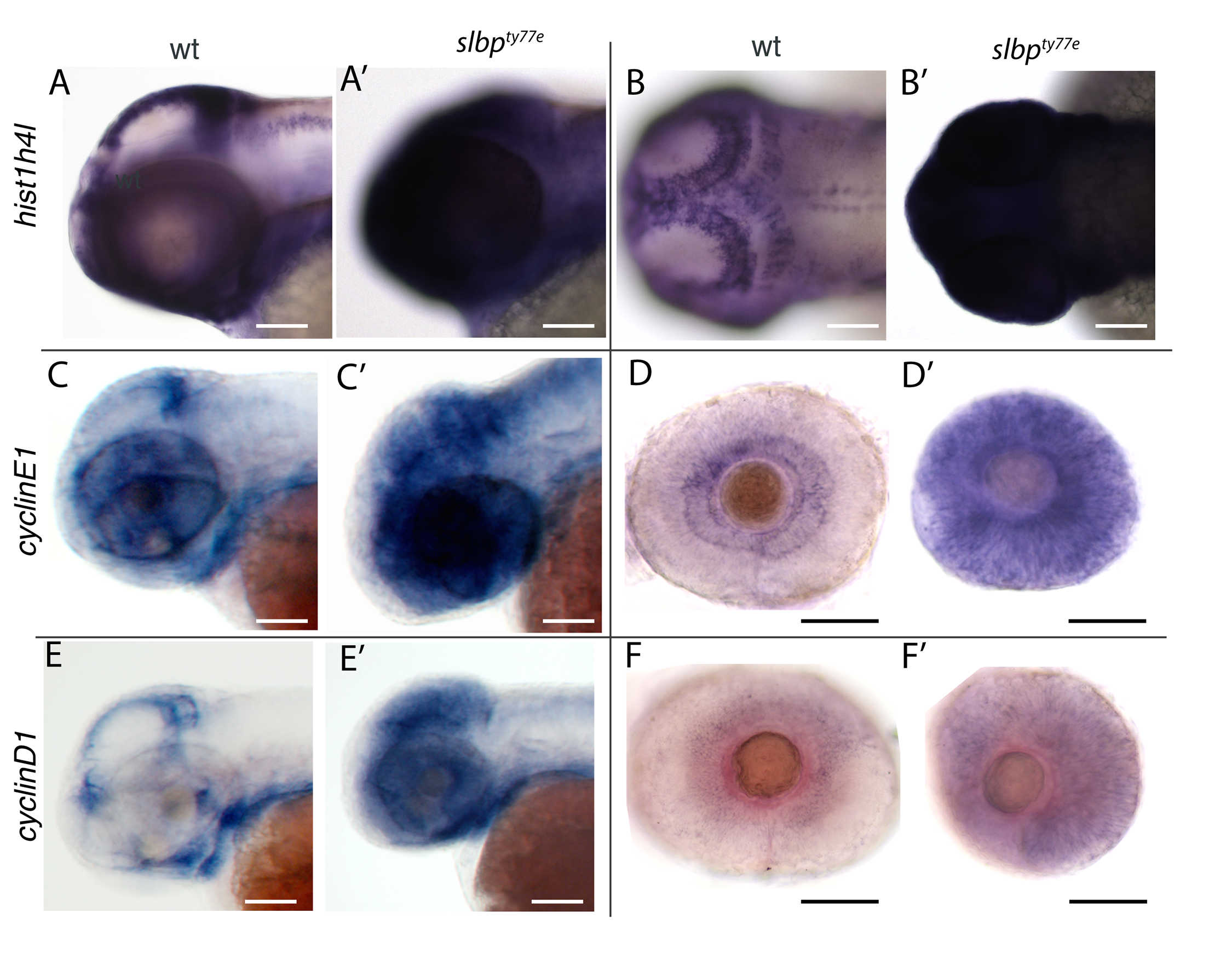

Supplement: S3 Fig — Images of wildtype (wt, A-F) and slbpty77e (A’-F’) heads (A-C’; E,E’) and eyes (D-F’) at 60hpf showing expression of genes indicated to the left of each row. Genotypes indicated at top of each column. Lateral (A,A’; C-F’) and dorsal view (B,B’). Scale bars: 100μm. (TIF) [file pone.0211073.s003.tif]
